# Supplementary material for: Exploring machine learning algorithms in sickle cell disease patient data: A systematic review
Source: PLoS One. 2024 Nov 11;19(11):e0313315. doi: 10.1371/journal.pone.0313315 (PMC11554206; doi:10.1371/journal.pone.0313315)
Supplement: S2 File — (DOCX) [file pone.0313315.s002.docx]

**Supporting information 2. List of articles included in the review and List of all titles returned (including repeats)**

**The exact syntax used for each database**

1. **Scopus**
   TITLE-ABS-KEY("sickle cell disease" OR "anemia sickle cell") AND TITLE-ABS-KEY("artificial intelligence" OR "machine learning") AND PUBYEAR > 2018
2. **Web of Science**
   ALL=((sickle cell disease OR sickle cell anemia) AND (artificial intelligence OR machine learning))
3. **MEDLINE/PubMed**
   ("sickle cell disease" OR "sickle cell anemia") AND ("artificial intelligence" OR "machine learning")
4. **IEEE Xplore**

("sickle cell disease" OR "sickle cell anemia") AND ("artificial intelligence" OR "machine learning")

*Observe the filter defined in the manuscript

**List of articles included in the review**

1. Abdulhay, Enas Walid, Ahmad Ghaith Allow, and Mohammad Eyad Al-Jalouly. "Detection of Sickle Cell, Megaloblastic Anemia, Thalassemia and Malaria through Convolutional Neural Network." 2021 Global Congress on Electrical Engineering (GC-ElecEng). IEEE, 2021.
2. Ayoade, Oluwafisayo Babatope, et al. "An Ensemble Models for the Prediction of Sickle Cell Disease from Erythrocytes Smears." EAI Endorsed Transactions on Pervasive Health and Technology 9 (2023).
3. Chy, Tajkia Saima, and Mohammad Anisur Rahaman. "A comparative analysis by KNN, SVM & ELM classification to detect sickle cell anemia." 2019 International conference on robotics, electrical and signal processing techniques (ICREST). IEEE, 2019.
4. de Haan, Kevin, et al. "Automated screening of sickle cells using a smartphone-based microscope and deep learning." NPJ digital medicine 3.1 (2020): 76.
5. Dheyab, Hamid Falah, et al. "Implementation a various types of machine learning approaches for biomedical datasets based on sickle cell disorder." 2020 4th International Symposium on Multidisciplinary Studies and Innovative Technologies (ISMSIT). IEEE, 2020.
6. Gollapalli, Mohammed, and Aljawharah Alfaleh. "An Artificial Intelligence Approach for Data Modelling Patients Inheritance of Sickle Cell Disease (SCD) in the Eastern Regions of Saudi Arabia." Mathematical Modelling of Engineering Problems 9.4 (2022).
7. Gollapalli, Mohammed, and Aljawharah Alfaleh. "Data Mining Hospital Treatment and Discharge Summary of Sickle Cell Disease Patients." 2023 International Conference on IT Innovation and Knowledge Discovery (ITIKD). IEEE, 2023.
8. Goswami, Neelankit Gautam, et al. "Sickle Cell Classification Using Deep Learning." 2023 3rd International Conference on Intelligent Technologies (CONIT). IEEE, 2023.
9. Goswami, Neelankit Gautam, et al. "Detection of sickle cell disease using deep neural networks and explainable artificial intelligence." Journal of Intelligent Systems 33.1 (2024): 20230179.
10. Güntürkün, Fatma, et al. "Using machine learning to predict rapid decline of kidney function in sickle cell anemia." EJHaem 2.2 (2021): 257-260.
11. Jennifer, Sanjeda Sara, et al. "Sickle cell disease classification using deep learning." Heliyon 9.11 (2023).
12. Ji, Yunhua, et al. "Identifying elevated risk for future pain crises in sickle-cell disease using photoplethysmogram patterns measured during sleep: a machine learning approach." Frontiers in digital health 3 (2021): 714741.
13. Mohammed, Akram, et al. "Using machine learning to predict early onset acute organ failure in critically ill intensive care unit patients with sickle cell disease: retrospective study." Journal of Medical Internet Research 22.5 (2020): e14693.
14. Padhee, Swati, et al. "Pain intensity assessment in sickle cell disease patients using vital signs during hospital visits." Pattern Recognition. ICPR International Workshops and Challenges: Virtual Event, January 10–15, 2021, Proceedings, Part II. Springer International Publishing, 2021.
15. Padhee, Swati, et al. "Improving pain assessment using vital signs and pain medication for patients with sickle cell disease: retrospective study." JMIR Formative Research 6.6 (2022): e36998.
16. Padrão, Eduardo Messias Hirano, et al. "Phenotypes of sickle cell intensive care admissions: an unsupervised machine learning approach in a single-center retrospective cohort." Annals of Hematology 101.9 (2022): 1951-1957.
17. Patel, Arisha, et al. "Machine‐learning algorithms for predicting hospital re‐admissions in sickle cell disease." British Journal of Haematology 192.1 (2021): 158-170.
18. Petrović, Nataša, et al. "Sickle-cell disease diagnosis support selecting the most appropriate machine learning method: Towards a general and interpretable approach for cell morphology analysis from microscopy images." Computers in Biology and Medicine 126 (2020): 104027.
19. Prashanthi, Gosika, and S. Pratap Singh. "Identification of Sickle Cell Anemia by Employing Hybrid Optimization and Recurrent Neural Network." 2023 3rd International Conference on Pervasive Computing and Social Networking (ICPCSN). IEEE, 2023.
20. Roy, Sumit Kumar, Saurabh Gupta, and Pankaj K. Jain. "Machine Learning-Based Disease Severity Prediction in Sickle Cell Patients: Spectroscopic Insights." PHOTOPTICS. 2024.
21. Sachdev, Vandana, et al. "A phenotypic risk score for predicting mortality in sickle cell disease." British journal of haematology 192.5 (2021): 932-941.
22. Sen, Bheem, et al. "Deep Learning based diagnosis of sickle cell anemia in human RBC." 2021 2nd International Conference on Intelligent Engineering and Management (ICIEM). IEEE, 2021.
23. Singh, Bikesh Kumar, and Hardik Thakkar. "Hydroxyurea Dosage Classification for Sickle Cell Disease Patients." 2021 6th International Conference on Inventive Computation Technologies (ICICT). IEEE, 2021.
24. Singh, Bikesh Kumar, et al. "Prediction of hydroxyurea effect on sickle cell anemia patients using machine learning method." Advances in Biomedical Engineering and Technology: Select Proceedings of ICBEST 2018. Springer Singapore, 2021.
25. Soni, Samiksha, Hardik Thakkar, and Bikesh Kumar Singh. "Transfer Learning for Sickle Cell Anemia and Trait Classification." 2022 Second International Conference on Power, Control and Computing Technologies (ICPC2T). IEEE, 2022.
26. Tengshe, Richa, et al. "Sickle cell anemia detection using convolutional neural network." 2021 12th International Conference on Computing Communication and Networking Technologies (ICCCNT). IEEE, 2021.
27. Vicent, Mabirizi, Kawuma Simon, and Safari Yonasi. "An algorithm to detect overlapping red blood cells for sickle cell disease diagnosis." IET Image Processing 16.6 (2022): 1669-1677.
28. Vuong, Caroline, et al. "Use of consumer wearables to monitor and predict pain in patients with sickle cell disease." Frontiers in Digital Health 5 (2023): 1285207.
29. Yeruva, S., et al. "Identification of Sickle Cell Anemia Using Deep Neural Networks. Emerg. Sci. J. 5, 200–210 (2021)."

**List of all titles returned (including repeats)**

1. A Stress and Pain Self-management mHealth App for Adult Outpatients With Sickle Cell Disease: Protocol for a Randomized Controlled Study
2. Preliminary evaluation of a mHealth coaching conversational artificial intelligence for the self-care management of people with sickle-cell disease
3. Can subjective pain be inferred from objective physiological data? Evidence from patients with sickle cell disease
4. Sickle cell disease classification using deep learning
5. Sickle cell anemia detection using convolutional neural network
6. SERS biosensing of Sickle cell hemoglobin from Normal hemoglobin
7. Machine Learning to Quantitate Neutrophil NETosis
8. Transfer Learning for Sickle Cell Anemia and Trait Classification
9. Recent studies and research on sickle cell disease: Statistical analysis and machine learning approach
10. Integrating deep learning with microfluidics for biophysical classification of sickle red blood cells adhered to laminin
11. Comparison of methods for early-readmission prediction in a high-dimensional heterogeneous covariates and time-to-event outcome framework
12. Text Mining on Hospital Stay Durations and Management of Sickle Cell Disease Patients
13. Machine learning-based approaches for identifying human blood cells harboring CRISPR-mediated fetal chromatin domain ablations
14. A Critical Review of Data Mining Techniques Used for the Management of Sickle Cell Disease
15. Building machine learning-based prediction system for critical diseases
16. Chest X-ray for pneumonia detection
17. Challenges and Opportunities of Precision Medicine in Sickle Cell Disease: Novel European Approach by GenoMed4All Consortium and ERN-EuroBloodNet
18. Respiratory resistance and reactance in adults with sickle cell anemia: Part 2—Fractional-order modeling and a clinical decision support system for the diagnosis of respiratory disorders
19. Using RBC shapes to distinguish between sickle cell disease and trait samples
20. Using Artificial Intelligence to Predict Surgical Shunts in Men with Ischemic Priapism
21. Using machine learning to predict early onset acute organ failure in critically ill intensive care unit patients with sickle cell disease: Retrospective study
22. Computing Sickle Erythrocyte Health Index on quantitative phase imaging and machine learning
23. Use of consumer wearables to monitor and predict pain in patients with sickle cell disease
24. Improved radiation expression profiling in blood by sequential application of sensitive and specific gene signatures
25. Identifying Elevated Risk for Future Pain Crises in Sickle-Cell Disease Using Photoplethysmogram Patterns Measured During Sleep: A Machine Learning Approach
26. An Application of Using Support Vector Machine Based on Classification Technique for Predicting Medical Data Sets
27. A statistical testing procedure for validating class labels
28. Machine Learning-Based Disease Severity Prediction in Sickle Cell Patients: Spectroscopic Insights
29. Predicting Pain in People With Sickle Cell Disease in the Day Hospital Using the Commercial Wearable Apple Watch: Feasibility Study
30. Multi-label Detection and Classification of Red Blood Cells in Microscopic Images
31. Prediction of hydroxyurea effect on sickle cell anemia patients using machine learning method
32. A phenotypic risk score for predicting mortality in sickle cell disease
33. Point-of-care microchip electrophoresis for integrated anemia and hemoglobin variant testing
34. Measuring Pain in Sickle Cell Disease using Clinical Text
35. Use of mobile health apps and wearable technology to assess changes and predict pain during treatment of acute pain in sickle cell disease: Feasibility study
36. Data Mining Hospital Treatment and Discharge Summary of Sickle Cell Disease Patients
37. Recent Artificial Intelligence Advances in Detection and Diagnosis of Sickle Cell Disease: A review
38. Hydroxyurea Dosage Classification for Sickle Cell Disease Patients
39. Selenium supplementation may improve COVID-19 survival in sickle cell disease
40. Haemoglobinopathies: A Review on Statistical Modelling Perspective (Haemoglobinopathies: Statistical Modelling Techniques)
41. RedTell: an AI tool for interpretable analysis of red blood cell morphology
42. Diagnostic value of spirometry vs impulse oscillometry: A comparative study in children with sickle cell disease
43. Blood cells detection using faster-RCNN
44. Paediatric Sickle Cell Detection using Deep Learning-A Review
45. Automated semantic segmentation of red blood cells for sickle cell disease
46. A novel deep learning approach for sickle cell anemia detection in human RBCs using an improved wrapper-based feature selection technique in microscopic blood smear images
47. Artificial intelligence in sickle disease
48. Classification of red cell dynamics with convolutional and recurrent neural networks: a sickle cell disease case study
49. Prospects of Machine Learning Algorithms in Healthcare Industry: A Review
50. An Artificial Intelligence Approach for Data Modelling Patients Inheritance of Sickle Cell Disease (SCD) in the Eastern Regions of Saudi Arabia
51. An Ensemble Models for the Prediction of Sickle Cell Disease from Erythrocytes Smears
52. Improving Pain Assessment Using Vital Signs and Pain Medication for Patients with Sickle Cell Disease: Retrospective Study
53. Identification of sickle cell anemia using deep neural networks
54. Machine Learning Predicts Acute Kidney Injury in Hospitalized Patients with Sickle Cell Disease
55. In vivo measurement of RBC survival in patients with sickle cell disease before or after hematopoietic stem cell transplantation
56. Artificial intelligence for improving sickle cell retinopathy diagnosis and management
57. Predictors of Diffusing Capacity in Children With Sickle Cell Disease: A Longitudinal Study
58. Deep learning-based cell identification and disease diagnosis using spatio-temporal cellular dynamics in compact digital holographic microscopy
59. Biophysical Profiling of Sickle Cell Disease Using Holographic Cytometry and Deep Learning
60. Digital Endpoints: Definition, Benefits, and Current Barriers in Accelerating Development and Adoption
61. Detection of sickle cell disease using deep neural networks and explainable artificial intelligence
62. A Machine Learning Model for Predicting Fetal Hemoglobin Levels in Sickle Cell Disease Patients
63. Continuous Pain Assessment Using Ensemble Feature Selection from Wearable Sensor Data
64. Phenotypes of sickle cell intensive care admissions: an unsupervised machine learning approach in a single-center retrospective cohort
65. Classification of red blood cell aggregation using empirical wavelet transform analysis of ultrasonic radiofrequency echo signals
66. Pain intensity assessment in sickle cell disease patients using vital signs during hospital visits
67. Algorithms for segmenting cerebral time-of-flight magnetic resonance angiograms from volunteers and anemic patients
68. Statistical Analysis of Hematological Parameters for Prediction of Sickle Cell Disease
69. Detection of Sickle Cell, Megaloblastic Anemia, Thalassemia and Malaria through Convolutional Neural Network
70. Wide-field imaging of sickle retinopathy
71. Trends in the Development of Diagnostic Tools for Red Blood Cell-Related Diseases and Anemias
72. Machine-learning algorithms for predicting hospital re-admissions in sickle cell disease
73. Spellchecking for the Story of Life with CRISPR-Cas9 and Base, Prime Editors
74. An algorithm to detect overlapping red blood cells for sickle cell disease diagnosis
75. Early detection of acute chest syndrome through electronic recording and analysis of auscultatory percussion
76. Sickle-cell disease diagnosis support selecting the most appropriate machine learning method: Towards a general and interpretable approach for cell morphology analysis from microscopy images
77. Predicting opioid dependence from electronic health records with machine learning
78. Revisiting prediction of collapse in hip osteonecrosis with artificial intelligence and machine learning: a new approach for quantifying and ranking the contribution and association of factors for collapse
79. Sickle Cell Classification Using Deep Learning
80. An AI-Based Model for the Prediction of a Newborn’s Sickle Cell Disease Status
81. Artificial intelligence in sickle disease
82. Artificial intelligence for improving sickle cell retinopathy diagnosis and management
83. Sickle cell disease classification using deep learning
84. Genetic regulation of fetal hemoglobin across global populations
85. Wide-field imaging of sickle retinopathy
86. Machine-learning algorithms for predicting hospital re-admissions in sickle cell disease
87. Biophysical Profiling of Sickle Cell Disease Using Holographic Cytometry and Deep Learning
88. Phenotypes of sickle cell intensive care admissions: an unsupervised machine learning approach in a single-center retrospective cohort
89. Measuring Pain in Sickle Cell Disease using Clinical Text
90. Machine Learning to Quantitate Neutrophil NETosis
91. Digital Endpoints: Definition, Benefits, and Current Barriers in Accelerating Development and Adoption
92. Deep learning-based cell identification and disease diagnosis using spatio-temporal cellular dynamics in compact digital holographic microscopy
93. Machine Learning Predicts Acute Kidney Injury in Hospitalized Patients with Sickle Cell Disease
94. A phenotypic risk score for predicting mortality in sickle cell disease
95. Pain Intensity Assessment in Sickle Cell Disease Patients Using Vital Signs During Hospital Visits
96. Use of consumer wearables to monitor and predict pain in patients with sickle cell disease
97. Computing Sickle Erythrocyte Health Index on quantitative phase imaging and machine learning
98. Predictors of Diffusing Capacity in Children With Sickle Cell Disease: A Longitudinal Study
99. Trends in the Development of Diagnostic Tools for Red Blood Cell-Related Diseases and Anemias
100. Predicting Pain in People With Sickle Cell Disease in the Day Hospital Using the Commercial Wearable Apple Watch: Feasibility Study
101. Machine learning-based approaches for identifying human blood cells harboring CRISPR-mediated fetal chromatin domain ablations
102. Improving Pain Assessment Using Vital Signs and Pain Medication for Patients With Sickle Cell Disease: Retrospective Study
103. Classification of red cell dynamics with convolutional and recurrent neural networks: a sickle cell disease case study
104. Point-of-care microchip electrophoresis for integrated anemia and hemoglobin variant testing
105. Sickle-cell disease diagnosis support selecting the most appropriate machine learning method: Towards a general and interpretable approach for cell morphology analysis from microscopy images
106. Revisiting prediction of collapse in hip osteonecrosis with artificial intelligence and machine learning: a new approach for quantifying and ranking the contribution and association of factors for collapse
107. A Stress and Pain Self-management mHealth App for Adult Outpatients With Sickle Cell Disease: Protocol for a Randomized Controlled Study
108. Using Artificial Intelligence to Predict Surgical Shunts in Men with Ischemic Priapism
109. Using machine learning to predict rapid decline of kidney function in sickle cell anemia
110. Preliminary Evaluation of a mHealth Coaching Conversational Artificial Intelligence for the Self-Care Management of People with Sickle-Cell Disease
111. Identifying elevated risk for future pain crises in sickle-cell disease using photoplethysmogram patterns measured during sleep: A machine learning approach
112. Can subjective pain be inferred from objective physiological data? Evidence from patients with sickle cell disease
113. RedTell: an AI tool for interpretable analysis of red blood cell morphology
114. Using Machine Learning to Predict Early Onset Acute Organ Failure in Critically Ill Intensive Care Unit Patients With Sickle Cell Disease: Retrospective Study
115. A statistical testing procedure for validating class labels
116. Predicting opioid dependence from electronic health records with machine learning
117. Diagnostic value of spirometry vs impulse oscillometry: A comparative study in children with sickle cell disease
118. The genetic dissection of fetal haemoglobin persistence in sickle cell disease in Nigeria
119. Respiratory resistance and reactance in adults with sickle cell anemia: Part 2-Fractional-order modeling and a clinical decision support system for the diagnosis of respiratory disorders
120. Classification of red blood cell aggregation using empirical wavelet transform analysis of ultrasonic radiofrequency echo signals
121. Use of Mobile Health Apps and Wearable Technology to Assess Changes and Predict Pain During Treatment of Acute Pain in Sickle Cell Disease: Feasibility Study
122. Integrating deep learning with microfluidics for biophysical classification of sickle red blood cells adhered to laminin
123. Improved radiation expression profiling in blood by sequential application of sensitive and specific gene signatures
124. Machine learning to optimize automated RH genotyping using whole-exome sequencing data
125. Algorithms for segmenting cerebral time-of-flight magnetic resonance angiograms from volunteers and anemic patients
126. Early Detection of Acute Chest Syndrome Through Electronic Recording and Analysis of Auscultatory Percussion
127. Longitudinal clinical data improve survival prediction after hematopoietic cell transplantation using machine learning
128. Design, Synthesis, and Antisickling Investigation of a Thiazolidine Prodrug of TD-7 That Prolongs the Duration of Action of Antisickling Aromatic Aldehyde
129. Comparison of methods for early-readmission prediction in a high-dimensional heterogeneous covariates and time-to-event outcome framework
130. Targeted modification of furan-2-carboxaldehydes into Michael acceptor analogs yielded long-acting hemoglobin modulators with dual antisickling activities
131. Continuous Pain Assessment Using Ensemble Feature Selection from Wearable Sensor Data
132. Association of Normal and Mutated APOL1 G2 Rs60910145 alleles with SCD, Body Mass Index, and Renal Function Biomarkers and Indices
133. Crowdsourced human-based computational approach for tagging peripheral blood smear sample images from Sickle Cell Disease patients using non-expert users
134. Measuring Pain in Sickle Cell Disease using Clinical Text
135. Hydroxyurea Dosage Classification for Sickle Cell Disease Patients
136. A Review of Automated Methods for the Detection of Sickle Cell Disease
137. Recent Artificial Intelligence Advances in Detection and Diagnosis of Sickle Cell Disease: A review
138. Text Mining on Hospital Stay Durations and Management of Sickle Cell Disease Patients
139. Implementation a Various Types of Machine Learning Approaches for Biomedical Datasets based on Sickle Cell Disorder
140. Data Mining Hospital Treatment and Discharge Summary of Sickle Cell Disease Patients
141. Automated Screening of Sickle Cells using a Smartphone-Based Microscope and Deep Learning
142. Detection of Sickle Cell, Megaloblastic Anemia, Thalassemia and Malaria through Convolutional Neural Network
143. A Comprehensive Review on Detection of Dacrocytes from Blood Smear Images using ML Algorithms
144. Early Detection of Acute Chest Syndrome Through Electronic Recording and Analysis of Auscultatory Percussion
145. Sickle cell anemia detection using convolutional neural network
146. Artificial Empathetic Intelligence for Leadership in Energy and Environmental Design Buildings
147. Comparative Analysis of Machine Learning Algorithms for Genomic Data
148. Machine Learning Algorithms in Healthcare: A Literature Survey
149. A Machine Learning Approach for Predicting Weight Gain Risks in Young Adults
150. Banana Disease Identification Using Machine Learning Based Technologies and Weather-Based Dispersion Analysis
151. A Review on Predicting Brain Stroke using Machine Learning
152. Threshold Based Feature Selection For Anemia Prediction
153. White Blood Cells Recognition and Classification using Convolutional Neural Network
154. A Machine Learning Approach to Detect the Brain Stroke Disease
155. Wearable-based Pain Assessment in Patients with Adhesive Capsulitis Using Machine Learning
156. Paediatric Sickle Cell Detection using Deep Learning - A Review
157. Monte Carlo method based model for augmenting data towards lubricant oil state analysis in heavy machine industry
158. Automatic pain assessment on cancer patients using physiological signals recorded in real-world contexts
159. A Multi-Class Classification Approach for Anemia Level Prediction with Machine Learning Models
160. Healthcare 4.0
161. Impact Analysis of the Complete Blood Count Parameter using Naive Bayes
162. Classification of Thalassemia Patients Using a Fusion of Deep Image and Clinical Features
163. Multi-label Detection and Classification of Red Blood Cells in Microscopic Images
164. Regression Based Machine Learning to Generate and Validate a Metric for Food Insecurity
165. Continuous Pain Assessment Using Ensemble Feature Selection from Wearable Sensor Data
166. A New Disease Candidate Gene Prioritization Method Using Graph Convolutional Networks
167. AI-Based Recipient Blood Type Matching Blood Transfusion Medical Device Design System
168. Advanced Blood Management System Community Method Based on Deep Learning
169. XAIA: An Explainable AI Approach for Classification and Analysis of Blood Anemia
170. Positive And Unlabeled Learning Algorithms And Applications: A Survey
171. Privacy-Aware Early Detection of COVID-19 Through Adversarial Training
172. Anemia Detection Through Image Analysis and Image Processing
173. White Blood Cell Segmentation Using DeepLabv3+ for Improved Hematological Disease Detection
174. Ocular Disease Recognition using Machine Learning
175. Pneumonia Detection Using Deep Learning Algorithms
176. Analysis of blood by Spectroscopy Near Infrared
177. Red Blood Cell Aggregation Classification Based on Ultrasonic Radiofrequency Echo Signals by An Improved Convolutional Neural Network
178. Potential of Raman Spectroscopy for Blood-Based Biopsy
179. Comparative Analysis of Risk Assessment Methods in StrokIndo Case Study
180. AF-SEG: An Annotation-Free Approach for Image Segmentation by Self-Supervision and Generative Adversarial Network
181. Emerging Technologies in the Field of Smart Monitoring Healthcare System with Cardiac Disease
182. Design, Fabrication, and Implementation of an Agriculture robot
183. Pain Detection from Facial Videos Using Two-Stage Deep Learning
184. Estimation of the incubation period of COVID-19 using boosted random forest algorithm
185. Blood Cells Detection Using Faster-RCNN
186. Modeling, Localization, and Segmentation of the Foveal Avascular Zone on Retinal OCT-Angiography Images
187. Implementation of Multiclass Algorithm for Sickle Cell Identification and Categorization – A Review
188. Caregiver Assessment Using Smart Gaming Technology: A Feasibility Study
189. Detection of Sickle Cell Anemia in Blood Smear using YOLOv3
190. Detection of Leukemia from Histopathological Image using Deep Learning Techniques
191. Objective Assessment of Beat Quality in Transcranial Doppler Measurement of Blood Flow Velocity in Cerebral Arteries
192. Semantic Segmentation of Anaemic RBCs Using Multilevel Deep Convolutional Encoder-Decoder Network
193. Sickle Cell Classification Using Deep Learning
194. “Deep Learning based diagnosis of sickle cell anemia in human RBC”
195. Transfer Learning for Sickle Cell Anemia and Trait Classification
196. A Comparative Analysis by KNN, SVM & ELM Classification to Detect Sickle Cell Anemia
197. A phenotypic risk score for predicting mortality in sickle cell disease
198. Using Machine Learning to Predict Early Onset Acute Organ Failure in Critically III Intensive Care Unit Patients With Sickle Cell Disease: Retrospective Study
199. Predicting Pain in People With Sickle Cell Disease in the Day Hospital Using the Commercial Wearable Apple Watch: Feasibility Study
200. Artificial intelligence in sickle disease
201. Phenotypes of sickle cell intensive care admissions: an unsupervised machine learning approach in a single-center retrospective cohort
202. Identifying Elevated Risk for Future Pain Crises in Sickle-Cell Disease Using Photoplethysmogram Patterns Measured During Sleep: A Machine Learning Approach
203. An algorithm to detect overlapping red blood cells for sickle cell disease diagnosis
204. Detection of sickle cell disease using deep neural networks and explainable artificial intelligence
205. Supervised Machine Learning Based Multi-Task Artificial Intelligence Classification of Retinopathies
206. Trends in the Development of Diagnostic Tools for Red Blood Cell-Related Diseases and Anemias
207. Machine Learning Predicts Acute Kidney Injury in Hospitalized Patients with Sickle Cell Disease
208. Artificial intelligence for improving sickle cell retinopathy diagnosis and management
209. Sickle cell disease classification using deep learning
210. Machine-learning algorithms for predicting hospital re-admissions in sickle cell disease
211. Hydroxyurea Dosage Classification for Sickle Cell Disease Patients
212. Measuring Pain in Sickle Cell Disease using Clinical Text
213. Detection of Sickle Cell, Megaloblastic Anemia, Thalassemia and Malaria through Convolutional Neural Network
214. Biophysical Profiling of Sickle Cell Disease Using Holographic Cytometry and Deep Learning
215. Sickle-cell disease diagnosis support selecting the most appropriate machine learning method: Towards a general and interpretable approach for cell morphology analysis from microscopy images
216. Use of consumer wearables to monitor and predict pain in patients with sickle cell disease
217. Preliminary Evaluation of a mHealth Coaching Conversational Artificial Intelligence for the Self-Care Management of People with Sickle-Cell Disease
218. Point-of-care microchip electrophoresis for integrated anemia and hemoglobin variant testing
219. Use of Mobile Health Apps and Wearable Technology to Assess Changes and Predict Pain During Treatment of Acute Pain in Sickle Cell Disease: Feasibility Study
220. The genetic dissection of fetal haemoglobin persistence in sickle cell disease in Nigeria
221. Diagnostic value of spirometry vs impulse oscillometry: A comparative study in children with sickle cell disease
222. Improving Pain Assessment Using Vital Signs and Pain Medication for Patients With Sickle Cell Disease: Retrospective Study
223. Predictors of Diffusing Capacity in Children With Sickle Cell Disease: A Longitudinal Study
224. Revisiting prediction of collapse in hip osteonecrosis with artificial intelligence and machine learning: a new approach for quantifying and ranking the contribution and association of factors for collapse
225. Using Artificial Intelligence to Predict Surgical Shunts in Men with Ischemic Priapism
226. Respiratory resistance and reactance in adults with sickle cell anemia: Part 2-Fractional-order modeling and a clinical decision support system for the diagnosis of respiratory disorders
227. SERS biosensing of Sickle cell hemoglobin from Normal hemoglobin
228. Machine learning in optical coherence tomography angiography
229. Improving concave point detection to better segment overlapped objects in images
230. Challenges and Opportunities of Precision Medicine in Sickle Cell Disease: Novel European Approach by GenoMed4All Consortium and ERN-EuroBloodNet
231. Predicting opioid dependence from electronic health records with machine learning
232. A novel deep learning approach for sickle cell anemia detection in human RBCs using an improved wrapper-based feature selection technique in microscopic blood smear images
233. Wide-field imaging of sickle retinopathy
234. Diagnosis and screening of abnormal hemoglobins
235. Advancing genome editing with artificial intelligence: opportunities, challenges, and future directions
236. Integrating deep learning with microfluidics for biophysical classification of sickle red blood cells adhered to laminin
237. Algorithms for segmenting cerebral time-of-flight magnetic resonance angiograms from volunteers and anemic patients
238. Machine Learning Predicts Acute Kidney Injury in Hospitalized Patients with Sickle Cell Disease
239. Machine Learning to Quantitate Neutrophil NETosis
240. Classification of red cell dynamics with convolutional and recurrent neural networks: a sickle cell disease case study
241. A Machine Learning Algorithm to Improve Risk Assessment for Patients with Sickle Cell Disease
242. Can subjective pain be inferred from objective physiological data? Evidence from patients with sickle cell disease
243. A Stress and Pain Self-management mHealth App for Adult Outpatients With Sickle Cell Disease: Protocol for a Randomized Controlled Study
244. Machine learning-based approaches for identifying human blood cells harboring CRISPR-mediated fetal chromatin domain ablations
245. Multi-label Detection and Classification of Red Blood Cells in Microscopic Images
246. Deep learning-based cell identification and disease diagnosis using spatio-temporal cellular dynamics in compact digital holographic microscopy
247. Individualized Prediction of Outcomes of Hematopoietic Cell Transplantation for Sickle Cell Disease: A Machine Learning Approach
248. Crowdsourced human-based computational approach for tagging peripheral blood smear sample images from Sickle Cell Disease patients using non-expert users
249. A New Strategy for the Morphological and Colorimetric Recognition of Erythrocytes for the Diagnosis of Forms of Anemia based on Microscopic Color Images of Blood Smears
250. Comparison of methods for early-readmission prediction in a high-dimensional heterogeneous covariates and time-to-event outcome framework
251. Use of Machine Learning to Predict 30-Day Reutilization of Care for Patients with Sickle Cell Disease Treated for Vaso-Occlusive Crisis
252. Early Detection of Acute Chest Syndrome Through Electronic Recording and Analysis of Auscultatory Percussion
253. Improved radiation expression profiling in blood by sequential application of sensitive and specific gene signatures
254. Design, Synthesis, and Antisickling Investigation of a Thiazolidine Prodrug of TD-7 That Prolongs the Duration of Action of Antisickling Aromatic Aldehyde
255. Analysis of coronavirus envelope protein with cellular automata model
256. Classification of red blood cell aggregation using empirical wavelet transform analysis of ultrasonic radiofrequency echo signals
257. Targeted modification of furan-2-carboxaldehydes into Michael acceptor analogs yielded long-acting hemoglobin modulators with dual antisickling activities
258. Low-Cost Automated Microscopy and Morphology-Based Machine Learning Classification of Sickle Cell Disease and Beta-Thalassemia in Nepal and Canada
259. AUGMENTING TRADITIONAL SICKLING TEST USING AUTOMATED MICROSCOPY AND MACHINE LEARNING: A LOW-COST APPLICATION FOR SICKLE CELL DISEASE SCREENING IN RURAL NEPAL
260. Quantitative optical coherence tomography angiography: A review
261. RedTell: an AI tool for interpretable analysis of red blood cell morphology
262. Association between priapism and HIV disease and treatment
263. Outcome of Severe Vaso-Occlusive Crisis in Sickle Cell Disease Adults Admitted to Referral Centers in Africa and Europe. Introduction of Machine Learning Methods to Improve the Presev Score
264. Application of machine learning for identification of heterotic groups in sunflower through combined approach of phenotyping, genotyping and protein profiling
265. Recognizing who is at risk for postpartum hemorrhage: targeting anemic women and scoring systems for clinical use
266. Feature preserving mesh network for semantic segmentation of retinal vasculature to support ophthalmic disease analysis
267. Identification of Small Molecules That Induce Therapeutic Levels of Fetal Hemoglobin for Treatment of Sickle Cell Disease By Pairing Machine Learning with High-Resolution Single Cell RNA Sequencing Maps of Adult and Fetal Human Erythropoiesis
268. Perspectives: on Precision Nutrition Research in Heart, Lung, and Blood Diseases and Sleep Disorders
269. Continuous Pain Assessment Using Ensemble Feature Selection from Wearable Sensor Data
270. A statistical testing procedure for validating class labels
271. CHANGE-seq reveals genetic and epigenetic effects on CRISPR-Cas9 genome-wide activity
272. Next-Generation Sequencing (NGS) and Third-Generation Sequencing (TGS) for the Diagnosis of Thalassemia
273. Mathematical modeling of regulatory networks of intracellular processes - Aims and selected methods
274. Association of Normal and Mutated APOL1 G2 Rs60910145 alleles with SCD, Body Mass Index, and Renal Function Biomarkers and Indices
275. Identification of common and divergent gene expression signatures in patients with venous and arterial thrombosis using data from public repositories
276. The road ahead in genetics and genomics
277. Genetic diversity fuels gene discovery for tobacco and alcohol use
278. The supply chain of migrant blood donors: an organisational interview study
279. Multi-ancestry transcriptome-wide association analyses yield insights into tobacco use biology and drug repurposing
