# Supplementary material for: Exploring machine learning algorithms in sickle cell disease patient data: A systematic review
Source: PLoS One. 2024 Nov 11;19(11):e0313315. doi: 10.1371/journal.pone.0313315 (PMC11554206; doi:10.1371/journal.pone.0313315)
Supplement: S3 File — (DOCX) [file pone.0313315.s003.docx]

**Supporting information 3. Risk of Bias.**

### **Risk of Bias**

| **Reference** | **Participant Selection and Data** | **Data Preprocessing** | **Algorithm Performance** | **Reporting of Results** |
| --- | --- | --- | --- | --- |
| Abdulhay et al. (2021) | Moderate | Moderate | Moderate | Moderate |
| Ayoade et al. (2023) | Moderate | Low | Low | Low |
| Chy et al. (2019) | High | Low | Moderate | Low |
| de Haan et al. (2020) | Moderate | Low | Low | Low |
| Dheyab et al. (2020) | Moderate | Moderate | Moderate | Low |
| Gollapalli & Alfaleh (2022) | Low | Low | Low | Low |
| Gollapalli & Alfaleh (2023) | Low | Moderate | Low | Low |
| Goswami et al. (2023) | Moderate | Moderate | Moderate | Low |
| Goswami et al. (2024) | Moderate | High | Moderate | Low |
| Güntürkün et al. (2021) | Low | High | Moderate | Low |
| Jennifer et al. (2023) | Moderate | High | Low | Low |
| Ji et al. (2021) | Moderate | Moderate | Moderate | Low |
| Mohammed et al. (2020) | Low | High | Low | Low |
| Padhee et al. (2021) | Moderate | Moderate | Low | Low |
| Padhee et al. (2022) | Low | Moderate | Low | Moderate |
| Padrão et al. (2022) | Low | Moderate | Low | Low |
| Patel et al. (2021) | Low | Low | Low | Low |
| Petrović et al. (2020) | Moderate | Low | Low | Moderate |
| Prashanthi & Singh (2023) | Low | Moderate | Moderate | Low |
| Roy et al. (2024) | High | Moderate | Moderate | Low |
| Sachdev et al. (2021) | Low | Low | Low | Moderate |
| Sen et al. (2021) | Moderate | Moderate | High | Moderate |
| Singh & Thakkar (2021) | Low | Moderate | Moderate | Low |
| Singh et al. (2021) | Moderate | Moderate | Low | Low |
| Soni et al. (2022) | High | Low | Low | Low |
| Tengshe et al. (2021) | Moderate | Low | Low | Moderate |
| Vicent et al. (2022) | Moderate | Moderate | Moderate | Moderate |
| Vuong et al. (2023) | High | Low | Low | Low |
| Yeruva et al. (2021) | Low | Moderate | Moderate | Moderate |

**Bias risk assessment**

| Risk of Bias Assessment for "Detection of Sickle Cell, Megaloblastic Anemia, Thalassemia and Malaria through Convolutional Neural Network"  Reference: Abdulhay, Enas Walid, Ahmad Ghaith Allow, and Mohammad Eyad Al-Jalouly. "Detection of Sickle Cell, Megaloblastic Anemia, Thalassemia and Malaria through Convolutional Neural Network." 2021 Global Congress on Electrical Engineering (GC-ElecEng). IEEE, 2021. |
| --- |
| Risk of Bias Summary:  Selection Bias:  The study used a dataset of 1815 microscopic images divided into training, validation, and holdout categories. The representativeness of the data is not explicitly discussed. Still, the collection of images from different conditions (malaria, megaloblastic anemia, sickle cell anemia, thalassemia, and regular blood cells) suggests an attempt to include a variety of cases. However, the lack of information about the origin and diversity of the samples may indicate a moderate risk of selection bias.  Rating: Moderate.  Preprocessing Bias:  Preprocessing included image normalization and division into datasets. There is no mention of class balancing techniques or missing data imputation, which can be a limitation, especially if there is an imbalance in the classes. Normalization is a good practice, but the absence of other techniques can increase the risk of bias.  Rating: Moderate.  Performance Bias:  The model was trained and validated using validation and holdout data, a best practice for evaluating model robustness and generalizability. However, cross-validation is not mentioned, which could strengthen the performance assessment. The reported accuracy of 93.4% is promising, but the lack of additional independent testing may indicate a moderate risk of performance bias.  Rating: Moderate.  Reporting Bias:  The report presents results, including the accuracy of the model. However, additional metrics such as sensitivity, specificity, or F-score, essential for a comprehensive assessment of model performance, are not provided. A lack of detail about the testing methodology and presentation of results can lead to a distorted interpretation of the results.  Rating: Moderate.  Overall Assessment of Risk of Bias: Based on the analyses, the study's risk of bias can be classified as moderate. Although the survey implemented several appropriate practices, the lack of detailed information on data representativeness, preprocessing techniques, and additional performance metrics contributes to this assessment. |

| Risk of Bias Assessment for "An Ensemble Models for the Prediction of Sickle Cell Disease from Erythrocytes Smears"  Reference: Ayoade, Oluwafisayo Babatope, et al. "An Ensemble Models for the Prediction of Sickle Cell Disease from Erythrocytes Smears." EAI Endorsed Transactions on Pervasive Health and Technology 9 (2023). |
| --- |
| Risk of Bias Summary:  Selection Bias:  The text does not provide detailed information about the representativeness of participant data or the selection criteria used. Although the data was obtained from a public erythrocytes IDB database, the study does not clearly detail the data.  Rating: Moderate.  Preprocessing Bias:  The text mentions performing preprocessing techniques such as Z-score normalization but does not provide sufficient detail on class balancing or missing data imputation. This information is important in the processes to avoid possible preprocessing bias. We consulted the database above and observed that it is balanced and has no missing values, but the authors do not mention these aspects in the text.  Rating: Low.  Performance Bias:  The use of cross-validation and comparison of algorithms are mentioned, but there is not enough information about the robustness and generalizability of the machine learning algorithms. The lack of independent testing of data sets may indicate a risk of performance bias despite being an underutilized detail.  Rating: Low.  Reporting Bias:  The text reports results comprehensively, with accuracy, sensitivity, specificity, F-score, and ROC curve metrics.  Rating: Low.  General Risk of Bias Assessment: Based on the analyses, the overall risk of bias of the text can be classified as low risk of bias. The lack of detailed information in several areas suggests that, although there is no clear evidence of bias, the transparency and robustness of the methods used could be improved. |

| Risk of Bias Assessment for "A comparative analysis by KNN, SVM & ELM classification to detect sickle cell anemia."  Reference: Chy, Tajkia Saima, and Mohammad Anisur Rahaman. "A comparative analysis by KNN, SVM & ELM classification to detect sickle cell anemia." 2019 International conference on robotics, electrical and signal processing techniques (ICREST). IEEE, 2019. |
| --- |
| Risk of Bias Summary:  Selection Bias:  The study uses a set of 80 images, 50 for training and 30 for testing. There is no detailed information about the representativeness of the images or the participant selection criteria. The lack of diversity in the samples may indicate a risk of selection bias. However, the risk can be considered low if the images represent the general population.  Rating: High.  Preprocessing Bias:  Preprocessing includes conversion to grayscale image, noise filtering, and image enhancement. The techniques used, such as the median filter and mask, are appropriate and common in image processing. There is no mention of class balancing or missing data imputation, but the approach appears robust.  Rating: Low.  Performance Bias:  The study compares three algorithms (KNN, SVM, and ELM) and provides performance metrics such as accuracy, sensitivity, specificity, and execution time. However, there is no mention of cross-validation or independent testing of datasets, which may limit the generalizability of the results.  Rating: Moderate.  Reporting Bias:  The results are presented clearly, with tables showing the algorithms' performance metrics. However, there is no discussion about limitations or possible sources of bias in the results, which could lead to a distorted interpretation. In any case, the results seem correct.  Rating: Low.  General Risk of Bias Assessment: Moderate. The study presents some good practices but also has areas that can be improved to reduce the risk of bias, especially in data selection and the robustness of performance tests. |

| Risk of Bias Assessment for "Automated screening of sickle cells using a smartphone-based microscope and deep learning."  Reference: de Haan, Kevin, et al. "Automated screening of sickle cells using a smartphone-based microscope and deep learning." NPJ digital medicine 3.1 (2020): 76. |
| --- |
| Risk of Bias Summary:  Selection Bias:  The study used 96 blood slides, of which 32 were from patients with sickle cell disease (SCD) and 64 from healthy individuals. Slides were obtained anonymously from a medical center, suggesting an effort to avoid selection bias. However, the sample's representativeness to the general population of patients with SCD is not discussed in detail. Therefore, although there is an effort to minimize bias, the lack of information about the sample's representativeness may indicate a risk of selection bias.  Rating: Moderate.  Preprocessing Bias:  The study mentions using a neural network to improve the quality of microscope images. Although balancing and missing data are not mentioned, the authors appear to have made an effort to avoid bias in preprocessing.  Rating: Low.  Performance Bias:  The algorithm's performance was evaluated using a separate validation dataset and blind tests on 96 slides, resulting in an accuracy of ~98% and an area under the curve (AUC) of 0.998. Using cross-validation and independent tests suggests that the risk of performance bias is low, as the performance metrics are robust and indicate good model generalization.  Rating: Low.  Reporting Bias:  The study presents comprehensive results, including relevant performance metrics such as accuracy and AUC. However, the lack of details about the data collection methodology and statistical analysis may raise concerns about the transparency of the results. Despite this, the presentation of key metrics suggests a low risk of reporting bias.  Rating: Low.  Overall Assessment of Risk of Bias: Considering the aspects discussed, the study presents a low risk of bias. Although efforts are made to minimize selection and performance bias, the lack of details on preprocessing and sample representativeness indicate areas that could be improved to increase the study's robustness and transparency. |

| Risk of Bias Assessment for "Implementation a various types of machine learning approaches for biomedical datasets based on sickle cell disorder."  Reference: Dheyab, Hamid Falah, et al. "Implementation a various types of machine learning approaches for biomedical datasets based on sickle cell disorder." 2020 4th International Symposium on Multidisciplinary Studies and Innovative Technologies (ISMSIT). IEEE, 2020. |
| --- |
| Risk of Bias Summary:  Selection Bias:  The study used a dataset collected from 1896 SCD patient samples from the Hemophilia Center at Alder Hey Children's Hospital, which suggests a representative sample of a specific population. However, representativeness may be limited if inclusion criteria are not broadly applicable to other SCD patient populations. A lack of information on the demographic diversity of participants may indicate a risk of selection bias.  Rating: Moderate.  Preprocessing Bias:  The text mentions the use of preprocessing techniques but does not provide specific details about how classes were balanced, whether normalization occurred, or how missing data was handled. The absence of information about these steps may indicate a risk of preprocessing bias, as these decisions may affect the effectiveness of the model.  Rating: Moderate.  Performance Bias:  The study presents a variety of machine learning algorithms and mentions the use of cross-validation and the division of data into training, validation, and test sets. However, the robustness and generalizability of the models are not discussed in depth, which may suggest a risk of performance bias. The lack of independent testing on external datasets also contributes to this assessment.  Rating: Moderate.  Reporting Bias:  Results are presented comprehensively, including performance metrics such as accuracy, AUC, sensitivity, and specificity.  Rating: Low.  Overall Assessment of Risk of Bias: Based on the analyses, the study has a moderate risk of bias. While there is a solid structure and clear presentation of results, gaps in information about sample representativeness, preprocessing techniques, model robustness, and reporting transparency indicate areas that could be improved to reduce the risk of bias. |

| Risk of Bias Assessment for "An Artificial Intelligence Approach for Data Modelling Patients Inheritance of Sickle Cell Disease (SCD) in the Eastern Regions of Saudi Arabia."  Reference: Gollapalli, Mohammed, and Aljawharah Alfaleh. "An Artificial Intelligence Approach for Data Modelling Patients Inheritance of Sickle Cell Disease (SCD) in the Eastern Regions of Saudi Arabia." Mathematical Modelling of Engineering Problems 9.4 (2022). |
| --- |
| Risk of Bias Summary:  Selection Bias:  Assessment of the representativeness of participant data indicates that the sample is predominantly composed of patients from Saudi Arabia, with a high percentage of individuals of Saudi nationality (84.82%) and a geographic distribution that includes several cities in the eastern region. However, including a limited number of participants from other nationalities may suggest a selection bias, as the sample may not reflect the full diversity of the population affected by the disease. Despite this, the sample is sufficiently large and appears to be representative of the local population in terms of demographic characteristics.  Rating: Low.  Preprocessing Bias:  The study mentions the application of preprocessing techniques such as normalization and imputation of missing data but does not provide sufficient detail on class balancing or the appropriateness of the techniques used. The lack of information about how data was handled prior to modeling may indicate a risk of preprocessing bias. However, the use of well-established machine learning algorithms suggests that, in general, preprocessing practices were followed.  Rating: Low.  Performance Bias:  The study uses cross-validation and compares multiple machine learning algorithms, which is a best practice to ensure the robustness and generalization of models. The analysis includes relevant performance metrics such as the percentage of correctly classified instances and the kappa statistic, which demonstrates an effort to evaluate the effectiveness of the models comprehensively.  Rating: Low.  Reporting Bias:  The results are presented comprehensively, including multiple performance metrics and a detailed analysis. Most of the relevant metrics appear to have been reported.  Rating: Low.  Overall Assessment of Risk of Bias: Based on the analyses, the study presents a low risk of bias overall, and the algorithms' performance is considered robust. |

| Risk of Bias Assessment for "Data Mining Hospital Treatment and Discharge Summary of Sickle Cell Disease Patients."  Reference: Gollapalli, Mohammed, and Aljawharah Alfaleh. "Data Mining Hospital Treatment and Discharge Summary of Sickle Cell Disease Patients." 2023 International Conference on IT Innovation and Knowledge Discovery (ITIKD). IEEE, 2023. |
| --- |
| Risk of Bias Summary:  Selection Bias:  Analysis of participant data reveals that the sample comprises 191,406 clinical records obtained from a hospital, which suggests a significant representation of the population of patients with Sickle Cell Disease (SCD) in the studied region. The large amount of data suggests a relatively low risk of selection bias.  Rating: Low.  Preprocessing Bias:  The study mentions using preprocessing techniques such as feature selection but does not provide sufficient detail on normalization, class balancing, or missing data imputation. The lack of information on how these aspects were handled may indicate a risk of bias, as the adequacy of preprocessing techniques is important for the validity of the results.  Rating: Moderate.  Performance Bias:  The machine learning algorithms used, such as Naive Bayes, SVM, and Neural Networks, were compared regarding accuracy and other performance metrics. The study mentions the use of cross-validation and parameter optimization, which suggests a robust approach to evaluating model generalization.  Rating: Low.  Reporting Bias:  The report presents a variety of performance metrics, including accuracy, sensitivity, and specificity, which are positive for the transparency of results. However, the absence of a more in-depth discussion of the methods' and results' limitations may lead to a distorted interpretation of the data.  Rating: Low.  Overall Assessment of Risk of Bias: Based on the assessments, the study has a low risk of bias in several areas. The representativeness of the sample and the transparency in the reports are positive points. Still, the need for more information about data selection and the results' robustness is evident. |

| Risk of Bias Assessment for "Sickle Cell Classification Using Deep Learning."  Reference: Goswami, Neelankit Gautam, et al. "Sickle Cell Classification Using Deep Learning." 2023 3rd International Conference on Intelligent Technologies (CONIT). IEEE, 2023. |
| --- |
| Risk of Bias Summary  Selection Bias:  The study uses a dataset from an open-source repository, including blood samples from healthy individuals and patients with sickle cell disease. The representativeness of the data appears adequate as it includes different categories of cells and is based on a previous study carried out at University College London (UCL). However, the description does not provide details about participant selection criteria, which may raise concerns about representativeness.  Rating: Moderate.  Preprocessing Bias:  The text mentions the use of normalization and class balancing techniques but does not provide specific details about how these techniques were implemented or whether they were appropriately applied to handle missing data. The lack of detailed information about preprocessing may indicate a risk of bias.  Rating: Moderate.  Performance Bias:  The study does not clarify whether it uses cross-validation and tests on independent data sets, as these are pre-trained transfer learning models. The reported accuracy of 93.88% and the comparison of different hyperparameter configurations suggest careful performance evaluation.  Rating: Moderate.  Reporting Bias:  The report presents comprehensive performance metrics, including precision, recall, and F1-score, which is positive for results transparency.  Rating: Low.  Overall Assessment of Risk of Bias: Considering the aspects discussed, the study presents a moderate risk of bias. While there are good practices in performance and reporting, concerns about data selection and preprocessing indicate that improvements can be made to increase study robustness and transparency. |

| Risk of Bias Assessment for "Detection of sickle cell disease using deep neural networks and explainable artificial intelligence."  Reference: Goswami, Neelankit Gautam, et al. "Detection of sickle cell disease using deep neural networks and explainable artificial intelligence." Journal of Intelligent Systems 33.1 (2024): 20230179. |
| --- |
| Risk of Bias Summary:  Selection Bias:  The study used a dataset from a public source, which may limit representativeness if the sample does not reflect the general population's diversity. However, the description of the data collection process and the inclusion of labeled images of sickled and non-sickle cells indicate an effort to minimize selection bias. The final sample was reduced to 1,664 images after manual inspection, which suggests care was taken in data selection. However, the description does not provide details about participant selection criteria, which may raise concerns about representativeness.  Rating: Moderate.  Preprocessing Bias:  The text leaves doubts about how the preprocessing occurred and does not provide specific details about how the techniques were implemented or whether they were adequately applied to deal with unbalanced and missing data. The lack of detailed information about preprocessing may indicate a risk of bias.  Rating: High.  Performance Bias:  The study presents a comparison between three deep learning models (GoogLeNet, ResNet-18 and ResNet-50) and mentions the use of transfer learning, which is a common practice to improve the robustness and generalization of models. However, there is no explicit mention of cross-validation or independent testing of datasets, which may limit the assessment of generalizability.  Rating: Moderate.  Reporting Bias:  The paper appears to report the results comprehensively, including performance comparisons between models and the introduction of XAI (Explainable Artificial Intelligence) to increase transparency.  Rating: Low.  Overall Risk of Bias Assessment: Based on the analyses, the study has a moderate risk of bias in several areas, primarily due to a lack of detailed information on preprocessing and performance validation. The representativeness of the data and the transparency in the reports are adequate but could be improved to strengthen the reliability of the results. |

| Risk of Bias Assessment for "Using machine learning to predict rapid decline of kidney function in sickle cell anemia."  Reference: Güntürkün, Fatma, et al. "Using machine learning to predict rapid decline of kidney function in sickle cell anemia." EJHaem 2.2 (2021): 257-260. |
| --- |
| Risk of Bias Summary:  Selection Bias:  The study evaluated 236 patients with SS and Sβ0 hemoglobin genotypes, with a mean age of 31.15 years and 56.4% women. The sample appears to be representative of the population of patients with sickle cell disease.  Rating: Low.  Preprocessing Bias:  The study mentions data imputation and predictive modeling techniques but does not provide sufficient detail on the suitability of preprocessing techniques such as class balancing and normalization. A lack of information about handling missing data may indicate a moderate risk.  Rating: High.  Performance Bias:  The machine learning models were evaluated using metrics such as AUC, sensitivity, and accuracy, and different datasets were used for validation. However, the description of cross-validation and the robustness of the algorithms is not sufficiently detailed, which may limit the generalization of the results.  Rating: Moderate.  Reporting Bias:  Results were reported comprehensively, including relevant performance metrics. Most metrics were presented clearly.  Rating: Low.  Overall Assessment of Risk of Bias: Considering the aspects discussed, the study presents a moderate risk of bias. Although participant selection and results reporting are adequate, the preprocessing and performance areas lack details that could increase confidence in the results. |

| Risk of Bias Assessment for "Sickle cell disease classification using deep learning."  Reference: Jennifer, Sanjeda Sara, et al. "Sickle cell disease classification using deep learning." Heliyon 9.11 (2023). |
| --- |
| Risk of Bias Summary:  Selection Bias:  The study used a dataset from the Erythrocytes IDB database, which contains images of blood samples from patients with sickle cell disease. The research appears to have used voluntary patient samples. However, there is not enough information about the selection criteria and the diversity of the sample.  Rating: Moderate.  Preprocessing Bias:  The study mentions data augmentation techniques and image normalization but does not provide further details about the risk of overfitting. The lack of information about the effect of synthetic data should be addressed in the text.  Rating: High.  Performance Bias:  The research used multiple machine learning models and conducted ablation experiments, which suggests a robust approach to evaluating model performance. Furthermore, performance metrics such as precision, recall, and F1-score were mentioned, and validation was performed on different models.  Rating: Low.  Reporting Bias:  The study presents comprehensive results, including several performance metrics, and discusses the results. However, transparency regarding the data selection process and the description of preprocessing techniques could be improved. Despite this, the presentation of the results is adequate, and there do not appear to be any significant distortions.  Rating: Low.  Overall Assessment of Risk of Bias: Based on the analyses, the study has a moderate risk of bias. Although there is a good structure and presentation of results, the areas of data selection, preprocessing, and model performance require more clarity and rigor to ensure the robustness and generalizability of the findings. |

| Risk of Bias Assessment for "Identifying elevated risk for future pain crises in sickle-cell disease using photoplethysmogram patterns measured during sleep: a machine learning approach."  Reference: Ji, Yunhua, et al. "Identifying elevated risk for future pain crises in sickle-cell disease using photoplethysmogram patterns measured during sleep: a machine learning approach." Frontiers in digital health 3 (2021): 714741. |
| --- |
| Risk of Bias Summary:  Selection Bias:  The study included 212 subjects, with a distribution of 36 individuals in the "high pain" category and 176 in the "low pain" category. The sample's representativeness can be questioned, since the selection was based on a single polysomnography (PSG) study per subject, which may not reflect the variability of health conditions over time. However, the research mentions that there is support for the stability of subject characteristics over time, as reported by Mullin et al. (p. 9).  Rating: Moderate.  Preprocessing Bias:  The study does not provide extensive details on the preprocessing techniques applied, such as class balancing or missing data imputation. Although cross-validation was used, the lack of information on handling missing data and normalization may indicate a risk of bias.  Rating: Moderate.  Performance Bias:  The use of k-fold cross-validation (k=4) was implemented, which is a suitable practice for evaluating the robustness of models. However, the small sample size and the possibility of "data leakage" during the validation process may compromise the generalization of the results.  Rating: Moderate.  Reporting Bias: The study presents a comprehensive comparison of performance metrics, including F1-score, precision, recall, and specificity. However, the description of some metrics and comparison with reference models could be more detailed. Despite this, the results are reported clearly and transparently.  Rating: Low.  Overall Assessment of Risk of Bias: Considering the aspects discussed, the study presents a moderate risk of bias. Sample selection and preprocessing techniques need more attention, while performance and reporting of results are more robust. |

| Risk of Bias Assessment for "Using machine learning to predict early onset acute organ failure in critically ill intensive care unit patients with sickle cell disease: retrospective study."  Reference: Mohammed, Akram, et al. "Using machine learning to predict early onset acute organ failure in critically ill intensive care unit patients with sickle cell disease: retrospective study." Journal of Medical Internet Research 22.5 (2020): e14693. |
| --- |
| Risk of Bias Summary:  Selection Bias:  The study presents a sample of 163 meetings with clearly defined inclusion and exclusion criteria. Data were collected from patients with sickle cell disease, and participant selection appears to be representative of the target population. The general selection approach is appropriate.  Rating: Low.  Preprocessing Bias:  The study used robust feature extraction techniques, including normalization and feature selection based on multiple hypothesis testing. Using a feature importance algorithm (Random Forests) to select the most discriminatory variables is a positive point. However, there is explicit mention of class imbalance, which may be relevant.  Rating: High.  Performance Bias:  The study applied 5-fold cross-validation and repeated testing to reduce bias, which is a best practice to ensure the robustness of models. Furthermore, the models were tested on independent data sets, increasing the results' generalizability. The comparison between different machine learning algorithms (MLP, SVM, RF, LR) is also a positive point.  Rating: Low.  Reporting Bias:  Results were reported comprehensively, including sensitivity and specificity metrics for each model. Including multimedia appendices to detail feature extraction and the importance of variables is a positive aspect.  Rating: Low.  Overall Assessment of Risk of Bias: Considering the aspects discussed, the study presents a low risk of bias, mainly due to participant selection and data preprocessing issues. Algorithm robustness and reporting transparency help mitigate some of these risks, but the overall approach is still room for improvement. |

| Risk of Bias Assessment for "Pain intensity assessment in sickle cell disease patients using vital signs during hospital visits."  Reference: Padhee, Swati, et al. "Pain intensity assessment in sickle cell disease patients using vital signs during hospital visits." Pattern Recognition. ICPR International Workshops and Challenges: Virtual Event, January 10–15, 2021, Proceedings, Part II. Springer International Publishing, 2021. |
| --- |
| Risk of Bias Summary:  Selection Bias:  Participant representation was considered adequate, as the study included 50 patients with SCD, collecting data during different hospital visits (inpatient, outpatient, and outpatient assessment). However, participant selection was not discussed in detail, which may raise concerns about the generalizability of the results.  Rating: Moderate.  Preprocessing Bias:  The study used several preprocessing techniques, including missing data imputation and normalization. However, there is not enough information about class balancing, which could impact model performance. The lack of details on the suitability of preprocessing techniques suggests a risk of bias.  Rating: Moderate.  Performance Bias:  The machine learning algorithms were evaluated with different performance metrics, and the study used cross-validation to ensure the robustness of the results.  Rating: Low.  Reporting Bias:  The study presents comprehensive and transparent results, including several performance metrics for the models tested. However, the comparison with previous studies could be more detailed to avoid distortions. Despite this, the data presentation is clear and informative.  Rating: Low.  Overall Assessment of Risk of Bias: Considering the aspects discussed, the study presents a moderate risk of bias, mainly due to concerns with participant selection, preprocessing techniques, and model performance. Transparency in reporting is a positive point that helps mitigate overall risk. |

| Risk of Bias Assessment for "Improving pain assessment using vital signs and pain medication for patients with sickle cell disease: retrospective study."  Reference: Padhee, Swati, et al. "Improving pain assessment using vital signs and pain medication for patients with sickle cell disease: retrospective study." JMIR Formative Research 6.6 (2022): e36998. |
| --- |
| Risk of Bias Summary:  Selection Bias:  Assessment of the representativeness of participant data indicates that the sample was drawn from electronic health records (EHR) of a specific hospital, which may limit the generalizability of results to other populations. However, including 33,000 records from 496 patients over five years suggests a sizeable sample.  Rating: Low.  Preprocessing Bias:  The study mentions using preprocessing techniques to deal with missing data. However, there is insufficient detail about class balancing, which may affect the data quality used. Therefore, the risk of preprocessing bias is assessed as moderate.  Rating: Moderate.  Performance Bias: The use of 5-fold cross-validation and comparison between represented and non-represented data indicates a robust approach to evaluating the performance of machine learning algorithms.  Rating: Low.  Reporting Bias:  The study comprehensively presents results, including performance metrics for pain scales and confusion matrices. However, accuracy is the only measure presented in the results.  Rating: Moderate.  Overall Risk of Bias Assessment: Based on the analyses, the study has an overall moderate risk of bias. Although significant efforts are being made to mitigate bias, limitations in data selection, preprocessing, and reporting indicate areas that can be improved to increase the robustness and generalizability of results. |

| Risk of Bias Assessment for "Phenotypes of sickle cell intensive care admissions: an unsupervised machine learning approach in a single-center retrospective cohort."  Reference: Padrão, Eduardo Messias Hirano, et al. "Phenotypes of sickle cell intensive care admissions: an unsupervised machine learning approach in a single-center retrospective cohort." Annals of Hematology 101.9 (2022): 1951-1957. |
| --- |
| Risk of Bias Summary:  Selection Bias:  The study was carried out in a single center, which may limit the representativeness of the data. However, including all patients with SCD admitted to the ICU between 1996 and 2020 suggests a comprehensive selection criterion. The sample of 125 admissions appears to reflect a variety of cases.  Rating: Low.  Preprocessing Bias:  The study used appropriate preprocessing techniques for clustering algorithms, such as normalization and Gower distance analysis for mixed data. However, there is no explicit mention of class balancing or missing data imputation, which may be a limitation.  Rating: Moderate.  Performance Bias:  The use of a hierarchical clustering approach and the evaluation of the optimal number of clusters through the silhouette plot indicate careful consideration of the model's robustness. However, the lack of cross-validation or testing on independent data sets may limit the generalizability of the results.  Rating: Low.  Reporting Bias:  The study presents results, including relevant performance metrics, such as hospital mortality and the distribution of cluster characteristics. However, the lack of detail about the statistical analysis and presentation of all performance metrics can lead to limited interpretation of the results.  Rating: Low.  Overall Assessment of Risk of Bias: Considering the aspects analyzed, the study presents a low risk of bias in several areas, which suggests that the authors had concerns in all areas. |

| Risk of Bias Assessment for "Machine‐learning algorithms for predicting hospital re‐admissions in sickle cell disease."  Reference: Patel, Arisha, et al. "Machine‐learning algorithms for predicting hospital re‐admissions in sickle cell disease." British Journal of Haematology 192.1 (2021): 158-170. |
| --- |
| Risk of Bias Summary:  Selection Bias:  The study sample included 446 Sickle Cell Disease (SCD) patients with unplanned hospitalizations, totaling 3,299 admissions. Participant selection seemed appropriate as data were collected from a representative set of SCD patients.  Rating: Low.  Preprocessing Bias:  The study used appropriate preprocessing techniques, such as missing data imputation and data normalization. The variables were carefully selected and represented, and one-hot encoding was applied to categorical variables. However, the description of class balancing techniques was not mentioned, which could be a limitation. Despite this, the overall approach seems robust.  Rating: Low.  Performance Bias:  The machine learning algorithms were evaluated using 100 different dataset splits to ensure the robustness and generalization of the models. The use of metrics such as C-statistic and precision-recall curves was also reported, indicating a rigorous performance evaluation.  Rating: Low.  Reporting Bias:  The study presented comprehensive results, including relevant performance metrics and clearly describing the methods used. However, transparency regarding all stages of the modeling process could be improved. Despite this, most of the information necessary to interpret the results was provided.  Rating: Low.  Overall Assessment of Risk of Bias: Based on the analyses, the study presents a low risk of bias in all categories evaluated. The methodologies used are robust and well-documented, contributing to the results' credibility. |

| Risk of Bias Assessment for "Sickle-cell disease diagnosis support selecting the most appropriate machine learning method: Towards a general and interpretable approach for cell morphology analysis from microscopy images."  Reference: Petrović, Nataša, et al. "Sickle-cell disease diagnosis support selecting the most appropriate machine learning method: Towards a general and interpretable approach for cell morphology analysis from microscopy images." Computers in Biology and Medicine 126 (2020): 104027. |
| --- |
| Risk of Bias Summary:  Selection Bias:  As indicated in the methodology, the study used an IDB public erythrocytes dataset, which collected data from samples of patients diagnosed with sickle cell disease. However, the study does not detail the data.  Rating: Moderate.  Preprocessing Bias:  The study mentions preprocessing techniques, such as dividing the data set into training and testing and applying cross-validation.  Rating: Low.  Performance Bias:  The study uses 10-fold cross-validation and an independent test set to evaluate the robustness of machine learning algorithms. Furthermore, the results are compared with state-of-the-art methods, suggesting a good model generalization. However, how the parameters were adjusted could be more detailed.  Rating: Low.  Reporting Bias:  The report presents comprehensive results, including performance metrics such as F-measure and SDS-score, and compares the results with other studies. However, the lack of detail in presenting all relevant metrics can be a limitation. Despite this, transparency in the presentation of results is a positive point.  Rating: Moderate.  Overall Assessment of Risk of Bias: Considering the aspects analyzed, the study presents a moderate risk of bias. Although model selection and performance are well addressed, gaps in preprocessing and presentation of results can impact the interpretation and applicability of findings. |

| Risk of Bias Assessment for "Identification of Sickle Cell Anemia by Employing Hybrid Optimization and Recurrent Neural Network."  Reference: Prashanthi, Gosika, and S. Pratap Singh. "Identification of Sickle Cell Anemia by Employing Hybrid Optimization and Recurrent Neural Network." 2023 3rd International Conference on Pervasive Computing and Social Networking (ICPCSN). IEEE, 2023. |
| --- |
| Risk of Bias Summary:  Selection Bias:  The study collected 340 red blood cell (RBC) images from multiple sources and used a labeling method to classify the images into three categories: normal, abnormal (sickle cell anemia), and other. The dataset's representativeness appears adequate as it includes a variety of cell types and conditions.  Rating: Low.  Preprocessing Bias:  Image preprocessing was used to remove noise and improve visualization, using techniques such as filtering and segmentation (Otsu Thresholding). Although the study mentions removing skews, it does not provide enough detail about class balancing or missing data imputation, which can impact the effectiveness of the model.  Rating: Moderate.  Performance Bias:  The study uses performance metrics such as precision, recall, and F1-score to evaluate the proposed system's effectiveness. However, there is no clear mention of utilizing cross-validation or independent testing on datasets, which is crucial to ensuring the robustness and generalizability of machine learning algorithms.  Rating: Moderate.  Reporting Bias:  The results are presented comprehensively, including tables and graphs comparing the proposed system's performance with that of existing approaches.  Rating: Low.  General Risk of Bias Assessment: The study presents a moderate risk of bias, mainly due to the lack of information about the algorithms' robustness and the preprocessing techniques' suitability. The data's representativeness is reasonable, but the lack of cross-validation and independent testing raises concerns about the generalizability of the results. |

| Risk of Bias Assessment for "Machine Learning-Based Disease Severity Prediction in Sickle Cell Patients: Spectroscopic Insights."  Reference: Roy, Sumit Kumar, Saurabh Gupta, and Pankaj K. Jain. "Machine Learning-Based Disease Severity Prediction in Sickle Cell Patients: Spectroscopic Insights." PHOTOPTICS. 2024. |
| --- |
| Risk of Bias Summary:  Selection Bias: The research used a dataset of 63 patients with sickle cell disease collected from Sickle Cell Institute Chhattisgarh, Raipur. The sample's representativeness is not explicitly discussed, but the selection of a single center and the number of patients may limit the generalization of the results.  Rating: High.  Preprocessing Bias: The study describes a preprocessing process that includes selecting relevant features and applying ANOVA to reduce dimensionality. Although it does not explicitly mention class balancing or missing data imputation, the general approach seems adequate. The lack of details about class balance may be a limitation, but it is not enough to indicate a significant bias.  Rating: Moderate.  Performance Bias: The research used 5-fold cross-validation to evaluate the effectiveness of machine learning models, which is a robust practice to ensure the generalizability of results. However, the amount of data for training and testing can lead to limited interpretations.  Rating: Moderate.  Reporting Bias: The study presents performance metrics such as accuracy, recall, precision, and specificity but does not provide a comprehensive discussion of all results or limitations.  Rating: Low.  Overall Assessment of Risk of Bias: Considering the aspects discussed, the study presents a moderate risk of bias. Although the research has appropriate approaches in several areas, the lack of detail in some sections and the limitation of the sample may impact the generalization and interpretation of the results. |

| Risk of Bias Assessment for "A phenotypic risk score for predicting mortality in sickle cell disease."  Reference: Sachdev, Vandana, et al. "A phenotypic risk score for predicting mortality in sickle cell disease." British journal of haematology 192.5 (2021): 932-941. |
| --- |
| Risk of Bias Summary:  Selection Bias:  The study included 600 patients with SCD, with a median age of 34 years and 51% female. The sample represents the SCD patient population, and the inclusion criteria are clear. There is no evidence of significant selection bias as the sample is sufficiently large and diverse.  Rating: Low.  Preprocessing Bias:  The study used a two-step machine learning (ML) method, including Random Survival Forests (RSF), to find missing data imputation and variable selection. No issues with class balancing or normalization were mentioned, but the imputation of missing data was addressed. The approach seems appropriate for the study's context.  Rating: Low.  Performance Bias:  The study used internal validation with Bootstrap and reported the performance of the models using C-statistics and Integrated Brier Score (IBS). Cross-validation was not explicitly mentioned, but internal validation suggests a robust approach. The generalizability of the results was not tested on independent data sets, which could be a limitation.  Rating: Low.  Reporting Bias:  The study presents comprehensive results, including performance metrics such as C-statistics and IBS, and discusses the importance of predictive variables. However, transparency regarding all aspects of the model could be improved, especially regarding potential limitations and selection bias.  Rating: Moderate.  Overall Assessment of Risk of Bias: The study presents a low risk of bias, attention to performance assessment, and the need for greater transparency in reporting. The selection and preprocessing approaches are robust, but the lack of validation on independent datasets and the need for more comprehensive reporting indicate areas for improvement. |

| Risk of Bias Assessment for "Deep Learning based diagnosis of sickle cell anemia in human RBC."  Reference: Sen, Bheem, et al. "Deep Learning based diagnosis of sickle cell anemia in human RBC." 2021 2nd International Conference on Intelligent Engineering and Management (ICIEM). IEEE, 2021. |
| --- |
| Risk of Bias Summary:  Selection Bias: The study uses a public IDB erythrocytes dataset. There is no detailed information about the participant selection criteria, which makes it challenging to assess representativeness and may introduce bias.  Rating: Moderate.  Preprocessing Bias: The study mentions the application of preprocessing techniques, such as image normalization and segmentation, but does not provide sufficient details about the techniques.  Rating: Moderate.  Performance Bias: Using five pre-trained models suggests a robust approach to evaluating model performance. However, the description of the models is not clear, which may impact the generalization of the results.  Rating: High.  Reporting Bias: The report presents relevant performance metrics, such as model accuracy, and appears comprehensive. However, the lack of details about the presentation of results and comparison with other studies may indicate a slight concern about transparency. Despite this, most metrics appear to be well documented.  Rating: Moderate.  Overall Assessment of Risk of Bias: Based on the analysis above, the study presents a high risk of bias. This is mainly due to selection and performance bias while preprocessing and reporting bias are moderate. The combination of these factors suggests that, although the study has merits, areas could be improved to ensure greater robustness and generalizability of the results. |

| Risk of Bias Assessment for "Hydroxyurea Dosage Classification for Sickle Cell Disease Patients."  Reference: Singh, Bikesh Kumar, and Hardik Thakkar. "Hydroxyurea Dosage Classification for Sickle Cell Disease Patients." 2021 6th International Conference on Inventive Computation Technologies (ICICT). IEEE, 2021. |
| --- |
| Risk of Bias Summary:  Selection Bias:  The study used a dataset of 1128 samples from SCD patients, suggesting a relatively large and potentially representative sample. However, the origin of the data (hospital in Liverpool, UK) may limit the generalizability of the results to other populations.  Rating: Low.  Preprocessing Bias:  The text mentions data splitting techniques, such as simple splitting into training and testing sets, but does not discuss normalization, class balancing, or missing data imputation. Lack of information about these techniques may indicate a risk of preprocessing bias.  Rating: Moderate.  Performance Bias:  The study compares the performance of two models (LSTM and ELM) and mentions the use of performance metrics but does not provide details on cross-validation or independent testing of datasets. The absence of this information may suggest a moderate risk of performance bias, as the robustness and generalizability of the models are not entirely guaranteed.  Rating: Moderate.  Reporting Bias:  The text reports the results comprehensively, including performance metrics such as accuracy, sensitivity, and F1 scores.  Rating: Low.  Overall Assessment of Risk of Bias: Based on the analyses, the study presents a moderate risk of bias in several areas, mainly due to the lack of details about preprocessing techniques, the robustness of the algorithms, and the presentation of results. Therefore, the overall risk of bias assessment is Moderate. |

| Risk of Bias Assessment for "Prediction of hydroxyurea effect on sickle cell anemia patients using machine learning method."  Reference: Singh, Bikesh Kumar, et al. "Prediction of hydroxyurea effect on sickle cell anemia patients using machine learning method." Advances in Biomedical Engineering and Technology: Select Proceedings of ICBEST 2018. Springer Singapore, 2021. |
| --- |
| Risk of Bias Summary:  Selection Bias:  The study used a dataset of 304 patients, but insufficient details were provided about the representativeness of the sample or participant selection criteria.  Rating: Moderate.  Preprocessing Bias:  The text mentions preprocessing techniques such as feature selection through filter and wrapper methods but does not provide details on normalization, class balancing, or missing data imputation. The absence of information on how these aspects were handled suggests a risk of preprocessing bias, as these techniques may affect the model's effectiveness.  Rating: Moderate.  Performance Bias:  The study used cross-validation and different classification methods, such as SVM, to evaluate the model's performance. Obtaining an accuracy of 94% is a positive indication.  Rating: Low.  Reporting Bias:  The report clearly and comprehensively presents performance metrics such as accuracy, sensitivity, and specificity. Therefore, the risk of reporting bias is considered low, but with some caveats.  Rating: Low.  Overall Assessment of Risk of Bias: Based on the analyses above, the study presents a moderate risk of bias. Although the results are clearly presented and machine learning techniques are used, the lack of detail on participant selection and preprocessing limits confidence in the conclusions. |

| Risk of Bias Assessment for "Transfer Learning for Sickle Cell Anemia and Trait Classification."  Reference: Soni, Samiksha, Hardik Thakkar, and Bikesh Kumar Singh. "Transfer Learning for Sickle Cell Anemia and Trait Classification." 2022 Second International Conference on Power, Control and Computing Technologies (ICPC2T). IEEE, 2022. |
| --- |
| Risk of Bias Summary:  Selection Bias:  The study uses a dataset consisting of 67 images of trait cases and 23 disease cases collected from a database prepared with the help of medical professionals. The sample's representativeness is limited due to the small number of cases, which may introduce selection bias. However, the data selection appears to have been done carefully, with validation by experts.  Rating: High.  Preprocessing Bias:  The text mentions that the study implemented the transfer learning technique with the AlexNet model, which does not require extensive preprocessing steps such as segmentation and feature extraction. The absence of explicit class balancing or normalization techniques can be a concern, but the transfer learning approach can mitigate some of these problems.  Rating: Low.  Performance Bias:  The study uses cross-validation with different data splitting protocols (hold-out, 5-fold, 10-fold) to evaluate the model's performance. The presentation of performance metrics, such as accuracy, sensitivity, and specificity, suggests a robust evaluation of the model.  Rating: Low.  Reporting Bias:  The article comprehensively describes the results, including various performance metrics and the ROC curve. The data presentation is precise and detailed.  Rating: Low.  Overall Assessment of Risk of Bias: Based on the analyses above, the study presents a moderate risk of bias. While some areas, such as preprocessing and reporting, are well covered, sample selection and the robustness of model performance can be improved to ensure more reliable and generalizable results. |

| Risk of Bias Assessment for "Sickle cell anemia detection using convolutional neural network."  Reference: Tengshe, Richa, et al. "Sickle cell anemia detection using convolutional neural network." 2021 12th International Conference on Computing Communication and Networking Technologies (ICCCNT). IEEE, 2021. |
| --- |
| Risk of Bias Summary:  Selection Bias: The study uses a public dataset (IDB erythrocytes) of 629 red blood cells (RBCs) images and mentions that the data selection was made from samples from patients with sickle cell anemia.  Rating: Moderate.  Preprocessing Bias: The study describes preprocessing techniques, such as histogram equalization and grayscale conversion, which are suitable for improving the quality of images before applying the machine learning model.  Rating: Low.  Performance Bias: The study uses 5-fold cross-validation to assess model robustness, a best practice to avoid overfitting and ensure generalization. Additionally, accuracy results are reported.  Rating: Low.  Reporting Bias: The study presents results comprehensively, including performance metrics such as accuracy, but does not provide a detailed analysis of all relevant metrics such as sensitivity and specificity. Lack of transparency regarding all results can lead to distortions in data interpretation.  Rating: Moderate.  General Risk of Bias Assessment: The study presents a moderate risk of bias. Although preprocessing and cross-validation techniques are adequate, the lack of comprehensive reporting on all performance metrics indicates areas that could be improved to reduce the risk of bias. |

| Risk of Bias Assessment for "An algorithm to detect overlapping red blood cells for sickle cell disease diagnosis."  Reference: Vicent, Mabirizi, Kawuma Simon, and Safari Yonasi. "An algorithm to detect overlapping red blood cells for sickle cell disease diagnosis." IET Image Processing 16.6 (2022): 1669-1677. |
| --- |
| Risk of Bias Summary:  Selection Bias: The study used a dataset of 1000 blood smear images obtained from a hematology atlas library, which suggests a systematic approach to data selection. However, there is no detailed information about the sample's representativeness of the general population of patients with sickle cell anemia. Therefore, there may be a risk of selection bias as the sample may not adequately reflect the diversity of the affected population.  Rating: Moderate.  Preprocessing Bias: The text mentions preprocessing techniques, such as removing unwanted components and applying edge detection. However, it does not provide a detailed description of normalization, class balancing, or missing data imputation.  Rating: Moderate.  Performance Bias: The algorithm was tested with different biometric characteristics and presented accuracy, specificity and sensitivity results. However, there is no mention of cross-validation or independent testing of datasets, which is important for evaluating the robustness and generalizability of the model.  Rating: Moderate.  Reporting Bias: The study presents results across different performance categories, but does not provide a comprehensive analysis of all relevant metrics. Lack of transparency regarding all results and lack of discussion about limitations can lead to distortions in reporting.  Rating: Moderate.  Overall Risk of Bias Assessment: Based on the analyses, the study's risk of bias is moderate. Although the study has a sizable database and utilizes image processing techniques, the lack of detail in several critical areas, such as sample representativeness, suitability of preprocessing techniques, validation of algorithm performance, and transparency in reports, contributes to this assessment. |

| Risk of Bias Assessment for "Use of consumer wearables to monitor and predict pain in patients with sickle cell disease."  Reference: Vuong, Caroline, et al. "Use of consumer wearables to monitor and predict pain in patients with sickle cell disease." Frontiers in Digital Health 5 (2023): 1285207. |
| --- |
| Risk of Bias Summary:  Selection Bias: The study included 19 patients with sickle cell disease (SCD) with a median age of 30 years, with most participants being HbSS. The sample is relatively small, but there is no information to suggest that the participant selection criteria were inappropriate or that the sample is not representative of the population of interest.  Rating: High.  Preprocessing Bias: The study used oversampling techniques to deal with class imbalance, assuming the pain score remained the same for up to 15 minutes before and after each recording. Although this approach may introduce some limitations, there is no evidence that the preprocessing techniques were inadequate. Rating: Low.  Performance Bias: The study used 10-fold cross-validation to assess the robustness of machine learning models, a best practice to ensure the generalizability of results. Models were compared with null models, and performance metrics were reported comprehensively.  Rating: Low.  Reporting Bias: The study presented comprehensive results, including performance metrics such as accuracy, F1-score, AUC, and RMSE. The information was reported clearly and transparently, without significant omissions.  Rating: Low.  Overall Assessment of Risk of Bias: Based on the analyses, the study has a moderate risk of bias. The methodologies used are appropriate, and the results are reported transparently, which contributes to the credibility of the study's conclusions. A wider range of data would improve the study. |

| Risk of Bias Assessment for "Identification of Sickle Cell Anemia Using Deep Neural Networks"  Reference: Yeruva, S., et al. "Identification of Sickle Cell Anemia Using Deep Neural Networks. Emerg. Sci. J. 5, 200–210 (2021)." |
| --- |
| Risk of Bias Summary:  Selection View: An assessment of the representativeness of participant data indicates that a sample of 1,387 patient records was obtained from a specific institution (Thalassemia and Sickle Cell Society), which may limit the generalizability of results. However, the sample appears to be appropriate for the study context, as it focuses on patients seeking diagnosis.  Rating: Low.  Preprocessing Bias: The study mentions the use of preprocessing techniques such as transforming labels into numeric types and dividing the dataset into training and testing. However, there is no clear mention of class balancing or missing data imputation, which could be a limitation.  Rating: Moderate.  Performance Bias: The study uses a dataset of 1387 samples and mentions implementing a Multilayer Perceptron (MLP) classifier with performance evaluation in terms of accuracy and log loss. However, there is no information about cross-validation, which may affect the robustness and generalizability of the results.  Rating: Moderate.  Reporting Bias: The study presents results clearly, including performance analyses such as accuracy and log loss. However, a lack of detail regarding the presentation of all relevant considerations can lead to a distorted interpretation of the results.  Rating: Moderate.  Overall Assessment of Risk of Bias: Based on the analysis above, the study presents a moderate risk of bias. Although there are some specific practices, limitations regarding preprocessing and reporting mean that improvements are warranted to increase the robustness and transparency of results. |
