# Supplementary material for: Exploring machine learning algorithms in sickle cell disease patient data: A systematic review
Source: PLoS One. 2024 Nov 11;19(11):e0313315. doi: 10.1371/journal.pone.0313315 (PMC11554206; doi:10.1371/journal.pone.0313315)
Supplement: Attachment — Submitted filename: Response to Reviewers.pdf [file pone.0313315.s006.pdf]

Rebuttal letter

Dear Editor,

We would like to express our sincere gratitude for the opportunity to submit the manuscript entitled "Exploring Machine Learning Algorithms in Sickle Cell Disease Patient Data: A Systematic Review" by Machado et al. for consideration in PLOS ONE. We are also profoundly grateful to the reviewers for their time and effort in analyzing our work in detail. Their detailed considerations and questions were extremely helpful in improving the manuscript. We prepared responses to each point raised by the reviewers.

To improve reproducibility, we included the exact syntax used to search for records in each database (see Supporting Information 2). This information has been added to the "Materials and Methods" section, specifically the "Search Strategy" subsection. The syntaxes for each database, including PubMed, Scopus, and others used, is now clearly specified allow other researchers to replicate our searches as recommended.

We present our considerations to the esteemed reviewers.

Responses to Reviewer #1:

*"The manuscript has an acronym problem. Acronyms can be useful, after begin defined, to refer to an idea in a more concise manner in subsequent regions of the manuscript. Or they can be completely avoided; the writing will be less concise, but readers don't need to remember any acronyms. *The authors need to decide which approach they want to use.* If they choose to use an acronym, it should be defined only once when the term is first mentioned. One problem example is that the phrase "sickle cell disease (SCD)" occurs 7 places in the paper. Similarly, "Support Vector Machine (SVM)" occurs nine times. Many other acronyms, especially for clinical metrics, are introduced but never used. An extreme case is "total of 14 features, such as weight, mean corpuscular volume (MCV), reticulocyte count (RETIC A), hemoglobin (Hb), reticulocyte count (RETIC%), HbF, platelets (PLTS), lactate dehydrogenase (LDH), neutrophils (white blood cells NEUT), alanine aminotransferase (ALT), bilirubin (BILI), aspartate aminotransferase (AST), bio-blood corporal (BIO)". These acronyms should be dropped to increase readability."*

Authors' response: Thank you for your valuable feedback regarding using acronyms in our manuscript. After a thorough review, we understand that overusing abbreviations, particularly for infrequent terms, can reduce the clarity of the text.

To improve readability, we implemented the following changes:

1. Reduced unnecessary acronyms: following your suggestion, we removed acronyms for metrics and terms mentioned only once or sparingly throughout the text.
2. Kept commonly used acronyms: We retained acronyms like "SCD" (sickle cell disease) and "SVM" (Support Vector Machine), as they are used frequently in the manuscript. In line with the advice, we ensured that each acronym is defined and used consistently after its first mention.

"Tables 1 and 2 are important parts of the manuscript, with a brief review of each study. They would be more informative, however, if they included some additional information. At present they include ML methods and metrics of success, but the studies are hard to compare because they're quite heterogeneous. For Table 1, please change the "Method" column to "Method/Data" by adding the type of data (such as "blood smear images") used by each study. That column should also include sample size (number of patients (and/or images)) for each study. Otherwise, one cannot attempt to determine whether the metrics of success are due to the method or the sample size (and if the more successful studies simply used more data). A more minor point: in the "Main Results" Column of Tables 1 and 2, please reduce the number of significant figures to 3; additional significant figures are not very meaningful."

Authors' response: We agree that Tables 1 and 2 could be more informative with the suggested changes. We have implemented the following changes in the "Method" column of Table 1:

1. Changed column name from "Method" to "Method/Data(n)" and added the type of data used in each study (e.g., blood smear images, clinical data, and others). We also included the sample size (number of patients and/or images) used in each study. We believe this provides a more complete picture so that readers can consider the influence of data type and volume on results.
2. Reduction in the number of decimal places in the "Main Results" column of Tables 1 and 2 to 3. We agree that more decimal places do not add to result interpretation.

At the end of the "Machine Learning Approaches for Diagnosis", please comment about the overall success of these methods. In other words, respond to questions like, Can machine learning be effectively used to diagnose SCD? Is the automated diagnosis of SCD using machine learning a solved problem? If not, what areas of this challenge still need the most work?

Authors' response: We thank the reviewer for raising these concerns about the overall success of machine-learning approaches in SCD diagnosis. We recognize that this is an essential discussion to provide a more comprehensive view of the current state of research and future potential. In response to the comment, we

added a paragraph at the end of the "Machine learning approaches for diagnosis" section.

For Table 2, please change the "Method" column to "Method/Data" by adding the type(s) of data (such as "PPG" or "vital signs") used by each study to build the ML classifier.

Authors' response: We thank the reviewer for this suggestion, which will make Table 2 more transparent and informative. In response to the comment, we have made the following changes:

1. Changed column name from "Method" to "Method/Data(n)".
2. Added to the column the data types used in each study for building the machine learning classifier, including examples such as "PPG", "vital signs", "clinical data," and other data types as applicable to each study.

"At the end of the "Machine Learning Approaches for Monitoring", please comment about the overall success of these methods. In other words, respond to questions like, Can machine learning be effectively used to monitor SCD? Is the automated monitoring of SCD using machine learning a solved problem? If not, what areas of this challenge still need the most work?"

Authors' response: We thank the reviewer for raising these important points about the effectiveness of machine-learning approaches in SCD monitoring. To address these aspects, we added a paragraph at the end of the section.

"In the "Discussion" section (lines 430-432), this statement needs explanation and/or correction:

"CNNs, especially when combined with transfer learning techniques, have performed better in diagnosing sickle cell disease than traditional algorithms."

How did the authors make this conclusion? What "traditional algorithms" do the authors mean? The statement should be modified to more clearly and correctly summarize the published studies."

Authors' response: We thank the reviewer for highlighting the need for greater clarity and precision in this Discussion section. We agree that the statement should be better justified and revised to reflect published studies accurately. In response to the comment, we have made the following changes:

1. Specification of “traditional algorithms”: The original version did not clearly define “traditional algorithms.” We revised the text to clarify that we were referring to commonly used machine learning algorithms that are considered simpler than CNNs, such as support vector machines, random trees,

K-nearest neighbors, logistic regression, and others, which have been widely used in previous studies for SCD diagnosis.

2. Revision of conclusion: We revised the statement to better reflect the findings of the reviewed literature. The statement indicates that several studies have shown that convolutional neural networks and transfer learning techniques perform superiorly in disease diagnosis tasks, including SCD, compared to commonly used algorithms, particularly on medical imaging datasets.

"Lines 450-452

This statement needs explanation and/or correction:

"Machine learning techniques can significantly improve sickle cell disease diagnosis and monitoring accuracy and speed, potentially reducing the burden on healthcare systems."

Was current SCD diagnosis and monitoring accuracy and speed discussed in the manuscript? The authors have shown that ML methods can effectively diagnose SCD, but they haven't discussed how this compares with current cost, speed, and reliability of diagnosis."

Authors' response: We appreciate the reviewer's bringing up this significant concern. Upon examining the excerpt mentioned, we acknowledge that the statement regarding the advantages of machine learning techniques over traditional methods for diagnosing and monitoring SCD lacked focus, context, and discussion in the manuscript. As a result, we removed the assertion about the enhanced cost, speed, and reliability of diagnosis.

"Lines 487-490

This paragraph can be removed, as it is beyond the scope of the current work. It is factually correct but just not relevant."

Authors' response: We thank the reviewer for pointing this out. We agree that the paragraph goes beyond the scope of the paper and, while factually correct, does not directly contribute to the main objectives of the manuscript. Therefore, as suggested, we have removed the paragraph from lines 487-490.

"Conclusion (last paragraph)

Add a mention of the value of increasing training dataset sizes. This is included in the abstract and should also appear here."

Authors' response: We thank the reviewer for raising that. We agree that increasing the size of training datasets is a crucial point that should be mentioned in the conclusion, especially to reinforce what was presented in the abstract. We have

changed the last paragraph and added the attention that should be given in future work to increasing and diversifying training datasets.

Responses to Reviewer #2:

"1. Is the manuscript technically sound, and do the data support the conclusions?

The manuscript must describe a technically sound piece of scientific research with data that supports the conclusions. Experiments must have been conducted rigorously, with appropriate controls, replication, and sample sizes. The conclusions must be drawn appropriately based on the data presented.

Reviewer #1: Yes

Reviewer #2: Partly"

Authors' response: We thank the reviewer for highlighting this issue and note that the review was "partially" positive. To address any concerns about technical soundness and data support for the detailed statements, we would like to clarify that we reviewed the manuscript to ensure that all cited work was described in the required level of detail, including methodologies and sample sizes, as suggested. In specific areas where the data or methods do not fully support the statements, as asked, we are reviewing and adjusting to ensure that all statements are adequately supported. We are keen to improve our revisions based on additional details provided by the reviewers if the current changes are still not satisfactory.

"4. Is the manuscript presented in an intelligible fashion and written in standard English?

PLOS ONE does not copyedit accepted manuscripts, so the language in submitted articles must be clear, correct, and unambiguous. Any typographical or grammatical errors should be corrected at revision, so please note any specific errors here.

Reviewer #1: Yes

Reviewer #2: No"

Authors' response: We regret that the manuscript did not fully meet the standards of clarity and correctness expected by the reviewer. To address your concerns, we reviewed the manuscript for typographical errors, grammatical errors, and clarification issues. We made the following corrections and improvements:

1. Typographical and Grammatical Errors: We identified and corrected typographical and grammatical errors, ensuring the text was error-free and more fluent.

2. Clarity and Coherence: We revised the manuscript to improve clarity and coherence, adjusting sentences and paragraphs to ensure the text is clear and easy to understand.
3. **Expert Review: We considered having the manuscript reviewed by an English language expert to ensure that all aspects of clarity and correctness are adequately met.**

We have included a revised version of the manuscript with the versions you mentioned. We appreciate your patience and understanding and are confident that the revisions met your expectations.

"Major comments:

1. In Page 2, could you be more specific as to the article eligibility? What is originality and results presentations? What criteria were applied to identify eligible articles? Some small sample studies need to be excluded. "

Authors' response: We appreciate your comment. After reviewing the section, we agreed to improve clarity regarding the eligibility of articles. We made the following additions and changes on page 2:

* reviewed the eligibility criteria applied to select articles, such as the originality of the studies, whether they were peer-reviewed, whether the article was complete and whether they presented results on the topic of SCD. In addition, we considered the articles' methodological quality and whether the study focused on sickle cell disease.

* reviewed what we understand by originality in the context of our review, which means studies where the primary data is not literature reviews. We also clarified that the types of results presented that we consider relevant should be related to the diagnosis or monitoring of SCD and not a parallel event.

* reviewed the included articles regarding the exclusion of small-sample studies. We understood that all selected articles presented a methodology appropriate to our protocol. We even mentioned in the discussion the limitation of generalization in very small samples. We created a file with the risk of bias, where each article is detailed individually (Supporting information 3). We ask that the reviewer consider providing us with additional information if they are unsatisfied with any of the included studies.

"2. Page 3, what is results reproducibility section about? What authors did for reproducibility? It is not clear. "

Authors' response: We thank the reviewer for the observation. After reviewing the manuscript, we decided to remove the section that was unclear about the reproducibility of the results found in the systematic review since it does not contribute to the main objectives of the manuscript.

"3. When summarizing studies in Table 1 and Table 2, could authors provide some more details about sample sizes and whether training-test cross-validations were used or not? And the cohort homogeneity/race-ethnicity could impact the performance of each ML tool. Without constructing valid cross-validation, very high accuracy ML results are less reliable."

Authors' response: We thank the reviewer for commenting on that. We agree that Tables 1 and 2 could be more informative with the suggested changes, and have implemented the following changes:

1. Sample Size Breakdown: We added information about the sample sizes used in each study, including the number of participants or images, as applicable. We believe this will provide a more complete picture so that readers can consider the influence of data type and volume on results;
2. Cross-Validations: We included information about whether training-test cross-validations were used in the studies in the discussion section and the risk of bias file (Supporting information 3);
3. Cohort Homogeneity: We did not discuss the homogeneity of cohorts in terms of race and ethnicity and how this may impact the performance of machine learning tools. The articles reported in the review did not provide this information, thus arguing that the discussion goes beyond the scope of the manuscript. Although there is strong evidence that it may impact performance, we were unable to do any analysis in this regard.

"4. When concluding CNN and transfer learning techniques, authors also need to note that whether each model was applied to external data (not training but external validation). Without external validations, reproducibility is very questionable."

Authors' response: We thank the reviewer for highlighting this issue. After reviewing the manuscript, we agree with the question of reproducibility of studies that did not perform external validation. The community is aware of researchers' difficulty obtaining data (reported in most articles). The ideal scenario would be for all articles to use validation with external data, encompassing different scenarios and challenging machine learning models in the issue of generalization. Unfortunately, this is not always possible. None of the articles included in the systematic review used external validation data. Even so, we addressed the reviewer's question in the discussion section and the study risk of bias file (Supporting information 3).

We want to reiterate that we have thoroughly reviewed the manuscript to ensure it complies with all PLOS ONE style requirements. Files have been renamed as recommended by the editor, and we have followed the templates to ensure that the format of the text, tables, figures, and reference list complies with the journal guidelines.

Again, we would like to thank the reviewers for their kind comments, which were essential to improving the quality and clarity of the manuscript. We are confident that our changes have resulted in an improved version that will meet the standards required by PLOS ONE.

Yours sincerely,

Tiago Machado (on behalf of all authors)
