# Supplementary material for: Exploring machine learning algorithms in sickle cell disease patient data: A systematic review
Source: PLoS One. 2024 Nov 11;19(11):e0313315. doi: 10.1371/journal.pone.0313315 (PMC11554206; doi:10.1371/journal.pone.0313315)
Supplement: Attachment — Submitted filename: JA_review_24.docx [file pone.0313315.s005.docx]

This manuscript provides a systemic review of ML tools that were applied to diagnosis/monitoring of sickle cell disease. Authors did conduct extensive research in summarizing available ML literatures, however, I have some concerns that need to be clarified through revision.

Major comments:

1. In Page 2, could you be more specific as to the article eligibility? What is originality and results presentations? What criteria were applied to identify eligible articles? Some small sample studies need to be excluded.
2. Page 3, what is results reproducibility section about? What authors did for reproducibility? It is not clear.
3. When summarizing studies in Table 1 and Table 2, could authors provide some more details about sample sizes and whether training-test cross-validations were used or not? And the cohort homogeneity/race-ethnicity could impact the performance of each ML tool. Without constructing valid cross-validation, very high accuracy ML results are less reliable
4. When concluding CNN and transfer learning techniques, authors also need to note that whether each model was applied to external data (not training but external validation). Without external validations, reproducibility is very questionable.
